# Underdevelopment of gut microbiota in failure to thrive infants of up to 12 months of age

**DOI:** 10.3389/fcimb.2022.1049201

**Published:** 2022-12-12

**Authors:** Mei Zhang, Dan Miao, Qi Ma, Tao Chen, Tuanmei Wang, Shuyuan Yan, Wendan Zhu, Fan Zhou, Jun He, Xiaoni Kuang

**Affiliations:** Changsha Hospital for Maternal and Child Health Care Affiliated to Hunan Normal University, Changsha, Hunan, China

**Keywords:** infant, failure to thrive (FTT), gut microbiota, microbial functions, developmental trajectory

## Abstract

Laboratory and clinical studies have revealed the importance of gut microbiota in children with severe pediatric pathological conditions such as severe acute malnutrition (SAM); however, under relatively milder conditions such as, failure to thrive (FTT), the role of the gut microbiota remains poorly characterized. Here, we analyzed stool samples from 54 subjects with a clinical diagnosis of failure to thrive (FTT), 49 preterm subjects with corrected normal growth (NFTT-pre), and 49 healthy subjects (NFTT) between 3-12 months of age using 16S rRNA gene sequencing. We observed that the clinical condition of FTT, age, head circumference, intrauterine growth restriction (IUGR), and feeding methods significantly affected gut microbiota. The microbiota age of subjects was significantly correlated with their anthropomorphic features, and the FTT subjects exhibited underdeveloped gut microbiota characterized by a significantly decreased microbiota-for-age Z-score (MAZ). The FTT and NFTT-pre groups exhibited an obvious disrupted developmental trajectory of gut microbiota across age, and the development of their alpha diversities and the observed OTU and Shannon indices were inadequate, particularly in subjects with FTT. Moreover, sequential colonization and enrichment of bacteria such as *Bacteroides*, *Bifidobacterium*, *Streptococcus* and most age-discriminatory bacterial taxa and their microbial functions were disorganized in FTT compared to that in NFTT. Our results revealed an underdevelopment of the gut microbiota in infants with failure to thrive that possesses potential clinical and practical importance.

## Introduction

Failure to thrive describes the clinical conditions of children with insufficient growth, insufficient weight gain, and/or inappropriate weight loss (Development, W.H.O., 2009; Shields et al., 2012; Al Nofal and Schwenk, 2013). In China (Li hui, 2002), failure to thrive was typically defined based on anthropometric measurements of children with Length-for-age Z-scores (LAZ) that were below two standard deviations (-2 s.d.) compared to the median defined by the World Health Organization (WHO) reference growth standards (Development, W.H.O., 2009), and 5.54-6.26% of children were affected by this condition (Li jiajianghui et al., 2021). The morbidity of failure to thrive varies and is associated with the development level of the city (Development, 2009; Li jiajianghui, 2021), thus leading epidemiologists to speculate that inadequate nutrition intake may be the origin (Ingo Scholler, 2012). Self-pathophysiological factors (National Guideline Alliance (UK), 2017; Homan, 2016) such as inborn errors of metabolism, problems with the gastrointestinal system, cystic fibrosis, diarrhea, and liver disease have also been considered as triggers for failure to thrive. Although therapeutic food interventions reduce mortality in children with severe acute malnutrition, incomplete restoration of healthy growth and obscured etiology remain serious problems (Ahmed et al., 1999; Ashraf et al., 2012).

Recent studies have proposed that the gut microbiota plays an important role in a broad range of metabolic disorders and diseases (Cani, 2019). Gut microbiota is tightly linked to host nutrient digestion and absorption (Backhed et al., 2004), energy utilization (Samuel et al., 2008), and lipogenesis (Backhed et al., 2004; Fei and Zhao, 2013). Zoological studies have revealed the importance of gut symbionts in the homeostasis of host metabolism. Germ-free mice exhibit impaired fat synthesis and weight gain difficulties and only survive on an amino acid-supplemented diet (Schaedler et al., 1965; Sommer and Backhed, 2013). Clinical studies have demonstrated distinct compositions of gut microbiota in subjects with obesity (Ley et al., 2006), diabetes (Qin et al., 2012), cystic fibrosis (Hayden et al., 2020), kidney disease (Joossens et al., 2019), SAM (Subramanian et al., 2014), and moderate acute malnutrition (MAM) (Raman et al., 2019) compared to the compositions of healthy individuals. In clinical practice, individual somatic conditions caused by microorganisms are relatively common such as *Salmonella* infection (Johari et al., 2019) and rabies (Hemachudha et al., 2013). In regard to the growth of children, a previous study revealed the development of the gut microbiota from infancy to childhood is spontaneous (Stewart et al., 2018) and synchronizes with the maturation of both the somatic body and nervous system. The majority of the studies focused on severe pediatric pathological conditions and observed a persistent immaturity of the gut microbiota in children with SAM (Subramanian et al., 2014). Immature gut microbiota was associated with abnormal growth phenotypes in both GF mice and pigs (Blanton et al., 2016), and this indicated the great impact of the gut microbiota on host growth. Using fecal microbiota transplantation (FMT) and microbiota-directed foods (the MDCF2) in both experimental animal models and in clinical volunteers, the causative and therapeutic roles of the gut microbiota in the context of SAM exhibited beneficial effects (Gehrig et al., 2019; Chen et al., 2021). Although progress has been made, the relationship between the occurrence of failure to thrive in children and the gut microbiota remains unknown.

To further explore the gut microbiota profile of infants with failure to thrive, 54 subjects with a clinical diagnosis of failure to thrive (FTT group), 49 preterm subjects (gestational age-corrected) with normal growth (NFTT-pre group), and 49 healthy subjects (NFTT group) between 3-12 months of postnatal age were recruited. Next-generation sequencing of the 16S rRNA gene was combined with clinical metadata to identify the effects of multiple factors such as age, region, sex, clinical comorbidity, feeding methods, and other factors on the gut microbiota of our cohort. In future clinical management of children with failure to thrive, the importance of re-recognizing the role of the gut microbiota in the etiology of failure to thrive is highlighted.

## Materials and methods

### Cohort description and study subjects

A total of 152 participants from the Hunan province of China were recruited for clinical diagnosis and treatment. All guardians provided informed consent for the collection of stool samples and trial information. This study was approved by the Research Ethics Committee of Changsha Hospital for Maternal and Child Health Care (NO:2020007). Written informed consent to participate in this study was provided by the legal guardians of the participants or their next of kin.

FTT subjects included 54 participants with a clinical diagnosis of failure to thrive that were recruited from our outpatient ward (age, 3–12 months) (**Table S1**). Failure to thrive was defined based on anthropometric measurements according to the World Health Organization (WHO) Child Growth Standards. Infants with length-for-age Z-scores (LAZ) of below two standard deviations (-2 s.d.) compared to the median of WHO reference growth standards were defined as FTT. Six subjects were preterm (gestational age < 37 weeks), and their gestational age-corrected LAZ was still below -2. Other anthropometric features such as weight-for-age Z-scores (WAZ) and head circumference-for-age Z-scores (HAZ) were also recorded. Participants who were finally diagnosed with inborn errors of metabolism, endocrine diseases, or congenital malformations were excluded.

Control subjects included a total of 98 age- and gender-matched infants with typical development. Of these, 49 subjects were preterm (NFTT-pre), and their current gestational age-corrected LAZ was at a healthy level (>-2). In clinical practice, growth of premature infants had the permissible “backward” age range and all the related anthropometric features of premature infants were corrected with gestational age to full term (40 week). Corrective gestational age for NFTT-pre and part of the FTT subjects = chronological age - (40 - gestational age)/4 (Prader et al., 1963; Jordan et al., 2005).

The metadata considered in this study are presented in **Table S1**. To register information, each sample was scored as 1 (yes) or 0 (no) for each factor. Comorbidities such as gastrointestinal problems, sleep complaints, and immune abnormalities refer to the body conditions that occurred in the past 2 weeks before sampling. None of the participants received any antibiotics 2 weeks prior to sampling. The usage of antibiotics and probiotics in the past 1 month prior to sampling was recorded. Infants who failed to add complementary foods at age of six were excluded.

### Fecal sample collection and DNA extraction

Fecal samples from each subject were obtained at home/outpatient/ward by their guardians as described in our previous study (Huang et al., 2021). In the outpatient ward, fecal samples were immediately removed into a storage kit (Zhejiang Hangzhou Equipment Preparation NO: 20190682) and transferred to a -80°C freezer. For samples collected by their guardians at home, storage kits containing dissolved stools were sent to our outpatient clinic (4°C) within 1 day and then stored at -80°C until further processing.

Microbial genomic DNA was extracted using a GHFDE100 DNA isolation kit (Zhejiang Hangzhou Equipment Preparation:20190952) following the manufacturer’s instructions. All of the resultant total DNA was quality controlled by gel electrophoresis and quantified using a Nanodrop spectrophotometer (Thermo 2000c). Samples with any organic change or incomplete/undetectable primary DNA bands (15 kb) were excluded.

### PCR amplification, quantification, and 16S rRNA gene sequencing

Sample-specific 7-bp barcodes were incorporated into the primers for multiplex sequencing. The V4 region of the 16S rRNA gene was PCR-amplified from microbial genomic DNA using specific primers (forward primer, 5’-GTGCCAGCMGCCGCGGTAA-3’; reverse primer, 5’-GGACTACHVGGGTWTCTAAT-3). The PCR components contained a total of 6 μL (10 μM) of primers, 25 μL of Phusion High-Fidelity PCR Master Mix (New England Biolabs, Ipswich, MA, USA), 10 μL of DNA template, and 6 μL of ddH2O. The PCR conditions were 98°C for 30 s, 25 cycles of 98°C for 15 s, 58°C for 15 s, and 72°C for 15 s, and a final extension at 72°C for 1 min. PCR amplicons were purified and quantified using Agencourt AMPure XP Beads (Beckman Coulter, Indianapolis, IN) and the PicoGreen dsDNA Assay Kit (Invitrogen, Carlsbad, CA, USA).

Amplicons were diluted to equal amounts and sequenced using the 2 × 150 bp paired-end read protocol on the Illumina HiSeq4000 platform at Guhe Info Technology Co. Ltd. (Hangzhou, China).

### Quality control and bioinformatics analysis of gut microbiota data

Barcodes equipped with raw sequences were assigned to the corresponding samples and identified as valid sequences. Inferior sequences were filtered using QIIME (Quantitative Insights into Microbial Ecology pipeline, v2020.6) (Caporaso et al., 2010) according to the following criteria: (i) sequences with a <150-bp length or <20 average Phred score; (ii) sequences that contained ambiguous bases or >8-bp mononucleotide repeats.

The average number of clean reads from each sample was 131,779. Qualified paired-end reads were blasted with each other, dereplicated (–derep_ full-length), and clustered (–cluster_unoise), and chimeras were filtered (–uchime3_denovo) using VSEARCH (V2.4.4) against the SILVA138 database. Sequences with similarity ≥97% were assembled into operational taxonomic units (OTUs) and discarded if the OTUs contained less than 0.001% of the total sequences across all samples.

Measurements of OTU-level alpha diversity such as Chao1, richness, abundance-based coverage estimator (ACE), and Shannon and Simpson indices of each sample were calculated using the OTU table in QIIME. Beta diversity analysis was performed to reveal the overall structural variation of gut microbial communities using Bray-Curtis distance metrics and was visualized *via* principal coordinate analysis (PCoA) (Ramette, 2007). The coordinates of the centroid of each group are the average values of the samples contained in the group on the specific PCoA axis (Raman et al., 2019). A phylogenetic tree with maximum likelihood at the genus level was constructed using phangorn (Schliep, 2011) (v.2.5.5) using a neighbor-joining tree as the starting point. Microbial functions were predicted using phylogenetic investigation of communities by reconstruction of unobserved states (PICRUSt) (Langille et al., 2013) and further analyzed using the Statistical Analysis of Metagenomic Profiles (STAMP) (Parks et al., 2014) software package (v2.1.3).

### Age-discriminatory bacterial taxa and microbiota age analysis

Random forest (RF) regression was used to select for age-discriminatory bacterial taxa (Subramanian et al., 2014). The relative abundances of bacterial taxa of the microbiota at the OTU level in time-series profiling of the NFTT subjects were extracted to regress against their chronological age by RF regression (default parameters, R package ‘RF’, ntree 1000, using default mtry). A report detailing the ranked lists of important taxa in order of reported feature importance was output after 100 iterations of the algorithm. A total of 30 taxa were selected to map the developmental spectrum of the gut microbiota in groups, and this model was then applied to subjects with FTT. The variance explanation for the model is 51%.

Multi-class convolutional neural network (CNNs) classification models were constructed based on the GUHE self-sequence cohort to further confirm microbiota age alterations in the FTT group. Briefly, the relative abundances of 75,489 OTU-level microbial taxa from 23,107 healthy subjects (aged 1 week to 102 years) were used as the training set. Three numerical matrices that included the genus level (summed abundance of the containing OTUs), OTU-level abundances, and correlations between the abundance of each genus from a total of 65,536 selected OTUs (the most abundant OTUs) were generated to output the 256 × 256 pixel diagram for subsequent CNNs classification. Only correlations greater than 0.75 were involved, and abundances of OTUs were logarithmically transformed (ranging from -1 to 1) and then normalized (ranged 0–255). Genera were positioned according to their abundance and correlation with the specific-paired genus. After positioning, OTUs belonging to the corresponding genera were shaded and filled according to their normalized abundances and outputted as a 256 × 256 pixel diagram. To maximize model efficiency, 23,107 subjects were divided into 52 subgroups according to their chronological age: 0-1y monthly; 1-2y every 2 months; 2-4y every 3 months; 4-10y yearly; 10-22y every 2 years; 22-40y every 3 years; 40-70y every 5 years; 70-102y every 8 years. Each subgroup contained at least 100 subjects. The corresponding diagrams were inputted to the CNNs against the sample chronological age using tensflow 1.13.1, trained by ResNet5050, and finally outputted as three predicted ages with the highest incidence. The microbiota age of each subject is the median of the three predicted ages. The performance of the adjusted CNNs model was evaluated in our validation set (another 8706 samples from GUHE) using MAE, and the R2 was 0.9554. Without any further parameter optimization, this model was applied to FTT- and NFTT-pre-children.

### Statistical analysis

Differences in anthropometric features between two or more groups were evaluated by one-way ANOVA followed by Dunnett’s test or Fisher’s protected least significant difference test using SPSS 24.0 (SPSS, Chicago, Illinois, USA). For gut microbiota features, Kruskal-Wallis tests followed by *post-hoc* Dunn tests with Benjamin-Holmes false discovery rate (FDR) correction were conducted. Mixed with all group, co-occurrence analysis was performed by calculating Spearman’s rank correlations between the microbial taxa/function and clinical features. Permutational Multivariate Analysis of Variance Using Distance Matrices (PERMANOVA) was performed by the “adonis” function in the R package “vegan” (http://CRAN.R-project.org/package=vegan) using Bray-Curtis distances with 1,000 permutations. EnvFit (He et al., 2018) was used to determine the effect size and significance of clinical or individual factors (covariates) on the gut microbiota.

## Results

### Anthropometric features in failure to thrive are related to the development of gut microbiota

**Figure 1:**
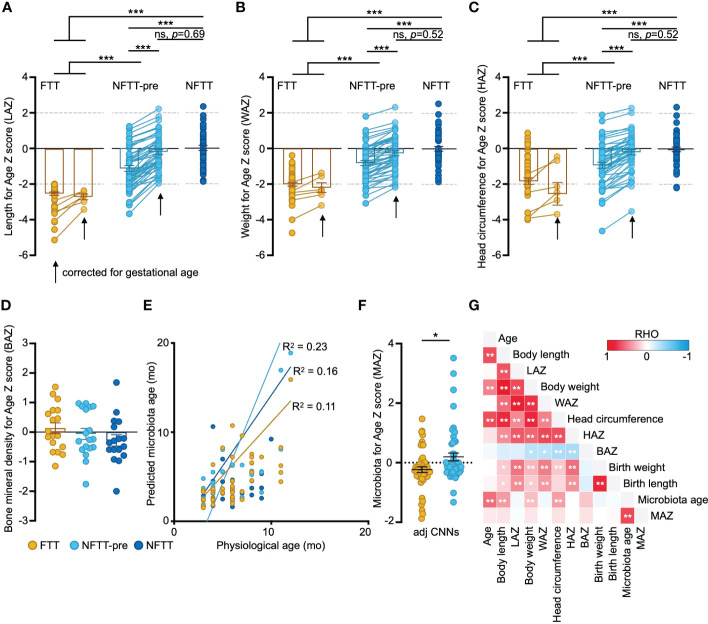
Anthropometric features in FTT are related to the development of gut microbiota. **(A-D)** Anthropometry features in FTT, NFTT-pre, and NFTT groups. Mean values ± SEM are plotted. One-way ANOVA, ***p < 0.001. **(E)**. Predictions of microbiota age in the FTT, NFTT-pre and NFTT subjects, respectively. Each circle represents an individual fecal sample, and the curves are smoothed linear fit between microbiota age and chronological age. **(F)** Microbiota for Age Z score (MAZ) in the FTT and NFTT-pre groups. Mean values ± SEM are plotted. One-way ANOVA, *p < 0.05. **(G)** The heatmap of the correlations between the gut microbiota developmental descriptive features and host anthropometry features. Based on Spearman’s correlation coefficient, *p < 0.05, **p < 0.01.

Fecal samples from 152 infants within 12 months of postnatal age were collected cross-sectionally. Full clinical information, including anthropometric, comorbidity, and recent medical, feeding, and perinatal data, is detailed in **Table S1**. A total of 54 infants with Length-for-age Z-scores of below -2 were clinically diagnosed with failure to thrive (FTT group), and 49 preterm but typically developed (gestational age-corrected Length-for-age Z-scores: 0 to -2, NFTT-pre group) and 49 healthy term infants (NFTT group) were defined as controls (**Figure 1A**). Six FTT subjects were preterm (FTT-pre), and 12 NFTT-pre subjects exhibited initial Length-for-age Z-scores of below -2 (**Table S1** and **Figure 1A**). The growth parameters within the neonatal period, the birth body length, and the weight were significantly decreased in the FTT group compared to these values in both the NFTT-pre and NFTT groups (**Table S2**). Proportions of stunted (Z score below -2), weight-for-age Z-scores (WAZ), and head circumference-for-age Z-scores (HAZ) in infants with failure to thrive (28 and 52% for WAZ and 12 and 26% for HAZ) were approximately 5- to 10-fold higher than was that of the NFTT-pre (5 and 10% and 10 and 20% for WAZ and HAZ, respectively) and NFTT (0 for WAZ and 2 and 4% for HAZ) (**Figures 1 B, C**) groups. Only a marginal increase (*p*=0.102) in bone mineral density-for-age Z-scores (BAZ) was observed in the FTT group (**Figure 1D**). Moreover, the incidence of gastrointestinal (GI) problems in the FTT and NFTT-pre groups was approximately 10 percent higher than was that in the NFTT group, while an increased tendency in the rates of infants with sleep complaints was observed in the NFTT-pre and NFTT groups (**Table 1** and **S2**).

**Table 1:** Distributions of comorbidities among 3 groups.

Comorbidity	FTT	NFTT-pre	NFTT
Any GI problem	16/54 (29.6%)	14/49 (28.5%)	10/49 (20.4%)
abdominal distention	3/54 (5.6%)	1/49 (2.0%)	1/49 (2.0%)
abnormal stool consistency	5/54 (9.3%)	3/49 (6.1%)	4/49 (8.2%)
constipation	0/54 (0.0%)	3/49 (6.1%)	1/49 (2.0%)
dyspepsia	3/54 (5.6%)	4/49 (8.2%)	2/49 (4.1%)
esophageal reflux	8/54 (14.8%)	3/49 (6.1%)	2/49 (4.1%)
Any sleep complaints	8/54 (14.8%)	8/49 (16.3%)	11/49 (22.4%)
difficulty falling asleep	0/54 (0.0%)	1/49 (2.0%)	2/49 (4.1%)
shallow sleep	2/54 (3.7%)	3/49 (6.1%)	2/49 (4.1%)
sleep fragmentation	6/54 (11.1%)	7/49 (14.3%)	9/49 (18.4%)
short sleep duration	0/54 (0.0%)	2/49 (4.1%)	1/49 (2.0%)
Food allergy	8/54 (14.8%)	2/49 (4.1%)	4/49 (8.2%)
Skin allergy	5/54 (9.3%)	1/49 (2.0%)	2/49 (4.1%)
Respiratory allergy	1/54 (1.9%)	0/49 (0%)	2/49 (4.1%)
Gastrointestinal allergy	0/54 (0.0%)	0/49 (0%)	0/49 (0%)

We then assessed the developmental state of the gut microbiota of each subject using a deep machine learning protocol (see Methods). For FTT and control groups, circles representing both the predicted microbiota age and chronological age of each sample could be diagonally distributed into the predicted microbiota age-chronological age coordinate axis (**Figure 1E**). The R^2^ of the fitting curves for FTT (0.11) was lower than were those of the NFTT-pre (0.23) and NFTT (0.16) groups. Although there was no statistical difference in the mean values of microbiota age among the three groups (**Figure S1A**), the mean microbiota-for-age Z scores (MAZ) in the FTT group (-0.23 ± 0.09, SEM) were significantly lower than were those of the NFTT-pre group (**Figure 1F**), thus indicating an obvious underdevelopment of gut microbiota in subjects with FTT. Similar trend of MAZ change was also found in the RF based prediction model (**Figure S1B**).

Consistently, infant LAZ was positively correlated with WAZ and HAZ but negatively correlated with the BAZ with statistical significance (**Figure 1G**). Birth weight and length were positively correlated with the subsequent body development level (such as the current LAZ, WAZ, and HAZ), and the microbiota age of infants in our cohort was significantly correlated with their body length, weight, and head circumference (**Figure 1G**).

### Multi-factorial impacts and disorganized development of gut microbiota in failure to thrive

**Figure 2:**
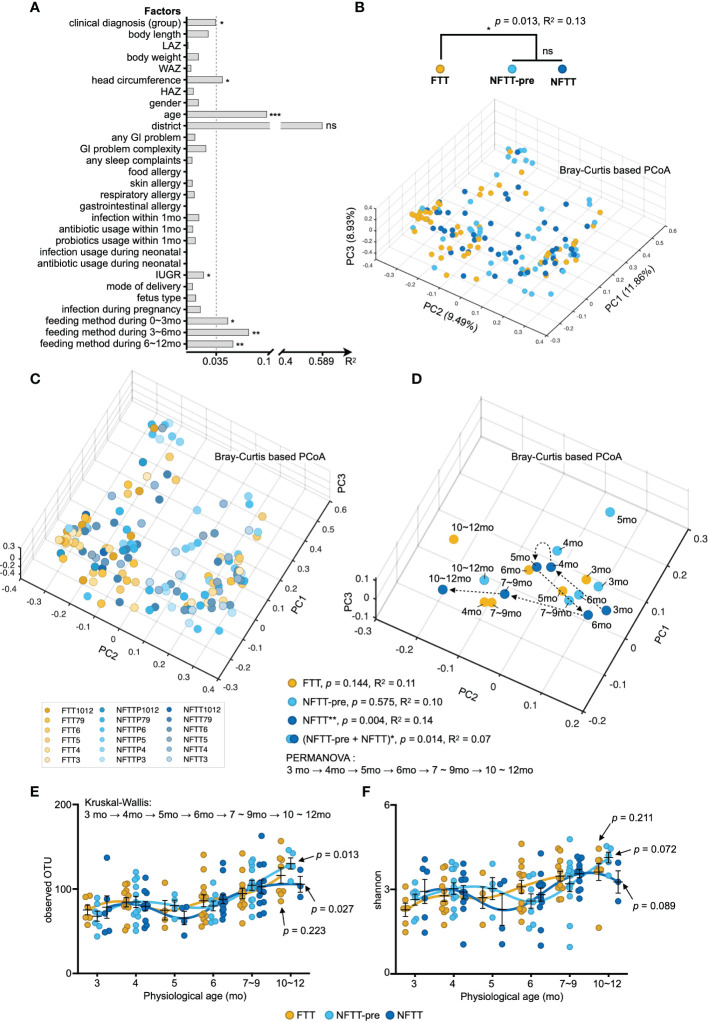
Multi-factorial impacts and disorganized development of gut microbiota in FTT. **(A)** Bars indicate the amount of effect size (R^2^) of each host factor on infant gut microbiota variations by EnvFit (vegan). Factors are roughly classified according to our metadata (**Table S1**), and the factors with significant effects are asterisked (FDR adjusted p value, *p < 0.05, **p < 0.01 and ***p < 0.001). **(B)** Three-dimensional diagram of principal component analysis (PCoA) based on OTU-level Bray–Curtis distance. Plots of each sample were dyed according to their clinical definition (group B) and chronological age **(C)**. **(D)** Each centroid indicates the cohort at a specific chronological age and is plotted on a PCoA space. Arrows indicate the temporal progression of microbiota reconfiguration in infants with chronological age. **(E, F)** Selected alpha diversity, observed OTU **(E)**, and Shannon **(F)** in the FTT, NFTT-pre, and NFTT groups. Mean values ± SEM are plotted. Kruskal-Wallis test with Benjamin-Holmes false discovery rate (FDR) correction. ns, no significance.

Previous studies have indicated that the human gut microbiota is affected by a wide range of host factors such as diet, genetics, disease, region, and medical conditions (He et al., 2018; Wan et al., 2019). Envfit (see Methods) was conducted to quantitatively evaluate the effect of individual infant factors on the gut microbiota. Five factors, including clinical diagnosis, head circumference, age, intrauterine growth restriction (IUGR), and feeding methods, from our metadata significantly affected infant gut microbiota (**Figure 2A**). In our cohort, infant chronological age contributed the most to the significance of gut microbiota and feeding methods (**Figure 2A**).

Principal component analysis (PCoA) based on the Bray-Curtis distance combined with PERMANOVA (also known as Adonis) testing revealed a significant difference (*p*=0.013) in the composition of the gut microbiota between the FTT group and the two control groups (**Figure 2B**). Although a previous study revealed that preterm birth may induce stunted gut microbiota in infants (Shao et al., 2019), there was no difference between the six preterm FTT subjects and other term FTT infants (**Figure S2A**). Additionally, we noticed that both the clinical conditions of FTT and age significantly affected gut microbiota, and we plotted each subject with gradient color according to their chronological age in a three-dimensional PCoA diagram (**Figure 2C**). Older infants were more likely to assemble in the negative axis of PC1 and 2 (**Figure 2C**). We then conducted PERMANOVA to infant gut microbiota against their age in different grouping methods. Using the arrow lines connecting the centroids of each group at a specific chronological age, an evolving temporal organization of gut microbiota with statistical significance was only formed in the NFTT group (*p*=0.004) (**Figure 2D**). Intriguingly, when the NFTT and NFTT-pre groups were combined, there was a significant difference in the developmental trajectory (PERMANOVA, *p*=0.014) of the mixed group (**Figure 2D**). For the profile of alpha diversity, the development of the observed OTU in NFTT and NFTT-pre was significant (**Figure 2E**), but the development of Shannon was more inadequate in FTT (**Figure 2F**).

### Colonization of gut microbiota in the FTT and NFTT-pre groups differs from that in NFTT group across age at different phylogenetic levels

**Figure 3:**
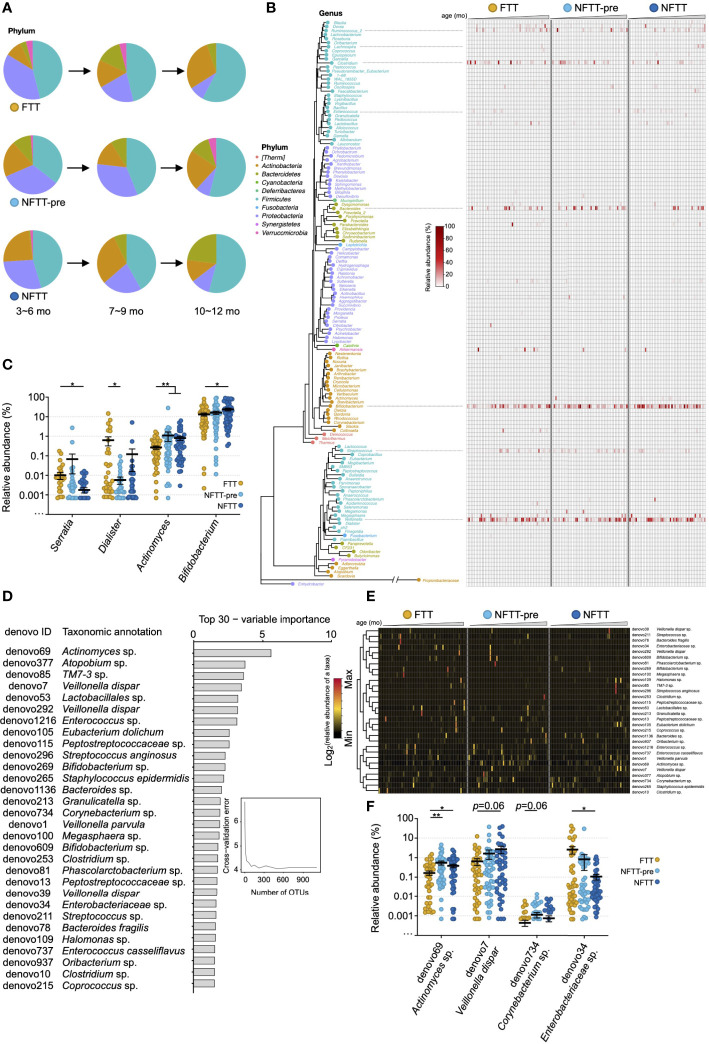
Colonization of gut microbiota in FTT and NFTT-pre groups differ from those of the NFTT group across age at different phylogenetic level. **(A)** Pie charts indicate the gut microbiota composition (mean relative abundance) at the phylum level across infant chronological age. **(B)** Neighbor joining phylogenetic tree of all of the 140 annotatable genera. Text indicates the generic name of each bacterial taxa (plot), and the color of the text and plot represents the level of phylum of each genus. Heatmap indicates the relative abundance of the specific genus in different groups. Samples are arranged from younger (left) to older (right). **(C)** Relative abundance of the 4 significant altered genera, including *Serratia*, *Dialister*, *Actinomyces* and *Bifidobacterium*, among the FTT, NFTT-pre and NFTT groups. Mean values ± SEM are plotted. Kruskal-Wallis test, *p < 0.05 and **p < 0.01. **(D)** A total of 30 age-discriminatory OTUs with the most variable importance identified *via* random forest regression of relative abundances of fecal bacterial taxa at the OTU level against infant chronologic age in NFTT subjects. The age-discriminatory OTU values are ranked in descending order of their importance to the accuracy of the model. **(E)** Heatmap indicates the relative abundance of the 30 most age-discriminatory bacterial taxa (OTU level) in the different groups. Samples are arranged from younger (left) to older (right). **(F)** Relative abundance of the 4 significant altered OTUs, including *Actinomyces* sp. (denovo69), *Veillonella dispar* (denovo7), *Corynebacterium* sp. (denovo734) and *Enterobacteriaceae* sp. (denovo34), among the FTT, NFTT-pre and NFTT groups. Mean values ± SEM are plotted. Kruskal-Wallis test, *p < 0.05 and **p < 0.01.

The composition of the gut microbiota was characterized at the multi-phylogenetic level. We deconstructed the developmental characteristics of the gut microbiota among the different groups at different taxonomic levels. Consistent with a previous study (Stewart et al., 2018; Roswall et al., 2021; Xiao et al., 2021), the main microbial communities at the phylum level in the infant gut primarily belonged to *Actinobacteria*, *Proteobacteria*, *Firmicutes*, and *Bacteroidetes* (**Figure 3A**). Bacteria belonging to *Proteobacteria*, *Firmicutes*, and *Actinobacteria* were prevalent and changed concomitantly with the infant age. Decreased *Proteobacteria* and increased *Bacteroidetes* were representative patterns of infant microbiota growth (**Figure 3A**). The relative abundance of *Actinobacteria* decreased in NFTT subjects across ages, but the FTT group exhibited the opposite trend (**Figure 3A**). The most prevalent genera among the three groups were *Bifidobacterium*, *Veillonella*, *Clostridium*, *Streptococcus*, *Megasphaera*, and *Ruminococcus_2*, and no group-specific genera were observed (**Figure 3B**). Interestingly, the order of colonization and development of many of the main bacterial genera exhibited large inter-group heterogeneity. *Bifidobacterium* was highly abundant among the majority of the NFTT infants, whereas in the FTT group only older infants could reach equal levels (**Figure 3B**). *Bacteroides* was only detected in older NFTT infants and were colonized in a disorderly manner in NFTT and NFTT-pre (**Figure 3B**). In contrast, the potential gastroenteric pathogen *Streptococcus* was detected in younger infants in the NFTT group and in older FTT and NFTT-pre infants (**Figure 3B**). Statistically, the relative abundance of *Serratia* decreased significantly in the NFTT group, and *Dialister* decreased significantly in the NFTT-pre group (**Figure 3C**). NFTT infants possessed more (with significance) *Bifidobacterium* compared to levels in the FTT subjects, and the proportions of *Actinomyces* increased significantly in both the NFTT-pre and NFTT groups (**Figure 3C**).

To better reveal the developmental characteristics of gut microbiota among the three groups, random forest (RF) regression was conducted (see method), and a list of the 30 most age-discriminatory bacterial taxa is presented in **Figure 3D**. Relative abundance changes of the 30 age-discriminatory bacterial taxa in the FTT group (left portion of the heatmap) across age were disordered, and the degree of the NFTT-pre group (middle portion of the heatmap) was relatively mild compared to that of the FTT group (**Figure 3E**). Consistent with the relative abundance change of *Actinomyces* (**Figure 3C**), the relative abundance of *Actinomyces* sp. (denovo69) increased significantly in both the NFTT and NFTT-pre groups (**Figure 3F**). The relative abundance of *Enterobacteriaceae* sp. (denovo34) exhibited a downward trend, particularly in the NFTT group (**Figure 3F**).

### Insufficient microbial functions enrichment in the FTT and NFTT-pre groups compared to those in the NFTT group

**Figure 4:**
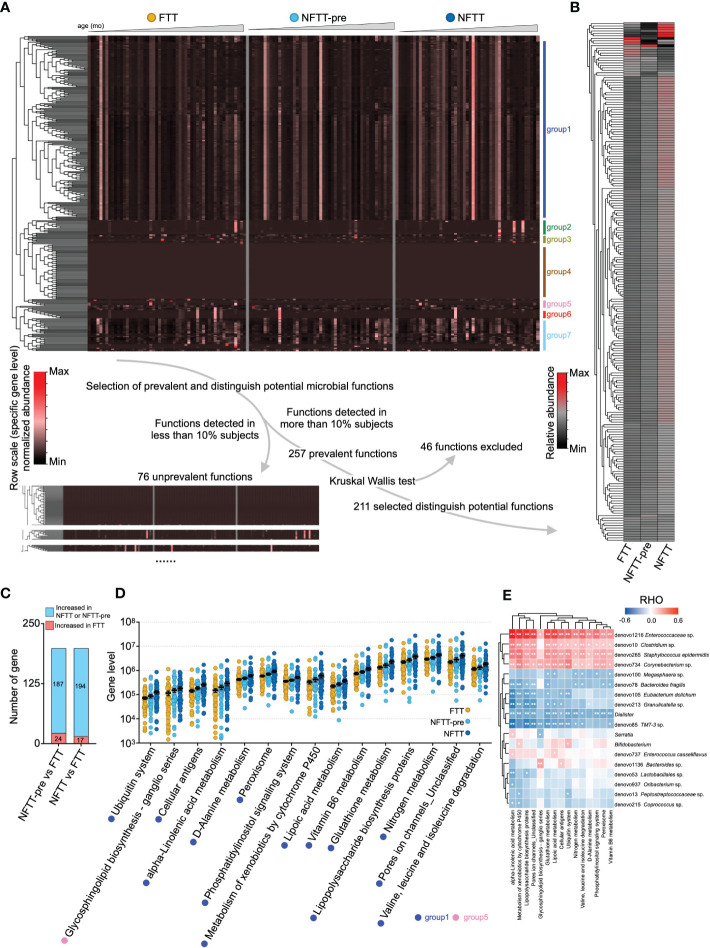
Insufficient microbial functions enrichment in the FTT and NFTT-pre groups compared to that in the NFTT group. **(A)** Overview of the gut microbial functions in the FTT, NFTT-pre and NFTT groups. Heatmap (above) indicates the relative abundance of the 333 microbial functions predicted using the PICRUSt against the KEGG profile. Samples are arranged from younger (left) to older (right), and the functions are clustered according the genetic sequential (by age) enrichment into 7 groups. Route (below) indicates the strategy for the selection of the 211 distinguished potential functions. **(B)** Relative abundance of the 211 distinguished potential functions in the FTT, NFTT-pre, and NFTT groups. **(C)** Brief statistical summary of the microbial functional changes between FTT and the other 2 groups. **(D)** Relative abundance of the 15 significant altered microbial functions across groups. Mean values ± SEM are plotted. Kruskal-Wallis test, *p < 0.05 and **p < 0.01. **(E)** The heatmap of the correlations between the gut microbial functions and the significant altered develop- or age-related microbial taxa (revealed in **Figure 3**). Based on Spearman’s correlation coefficient, *p < 0.05, **p < 0.01.

Using PICRUSt, 333 microbial metabolic functions were predicted and quantified utilizing the annotated KEGG profile (see Methods). These microbial functions could be clustered into seven groups (group 1-7) according to their relative abundance distributions across host chronological age changes (**Figure 4A**). To avoid the influence of rare microbial functions (with a very low number of carriers) on further analysis, 76 functions detected in less than 10% of subjects (16/76) were defined as ‘unprevalent’ functions and were excluded (**Figure 4A**). Additionally, 46 microbial functions that could not complete the Kruskal-Wallis test were excluded, and the remaining 211 functions were considered as distinguishing potential functions (**Figure 4A**). We further clustered microbial functions according to their abundance changes against age, and grouped the clusters with the same trends. The excluded microbial functions primarily belonged to Groups 2, 4, and 6 (clusters of microbial functions did not shift by age or only with extremely high/low at 1-2 age points). The mean relative abundance of 211 functions exhibited an upward trend in the NFTT and NFTT-pre groups (**Figure 4B**). In summary, 187 functions with increased mean abundance and 24 with decreased mean abundance were observed in the NFTT-pre group, and 194 functions with increased and 17 with decreased mean abundance in the NFTT group were observed (**Figure 4C**). Statistically, 15 microbial functions exhibited persistent upward abundance changes and increased significantly in the NFTT group compared to levels in the FTT group (**Figure 4D**). Fourteen of these belonged to group 1, and the remaining one belonged to group 5 (**Figures 4A, D**). Moreover, four taxa, including *Enterococcaceae* sp. (denovo1216), *Clostridium* sp. (denovo10), *Staphylococcus epidermidis* (denovo265), and *Corynebacterium* sp. (denovo734), exhibited significant positive correlations with almost all 15 significantly changed functions (**Figure 4E**). The genus *Dialister* was negatively related to 13 functions, and *TM7-3* sp. (denovo85) was negatively related to 11 functions with significance (**Figure 4E**). The early prevalence and significantly increased genera (in the NFTT group) and *Bifidobacterium* (**Figure 3B**) exhibited only positive correlations with the metabolism of xenobiotics by cytochrome P450 and the ubiquitin system (**Figure 4E**).

### Correlations between the gut microbial taxa/functions and infant anthropometric features

**Figure 5:**
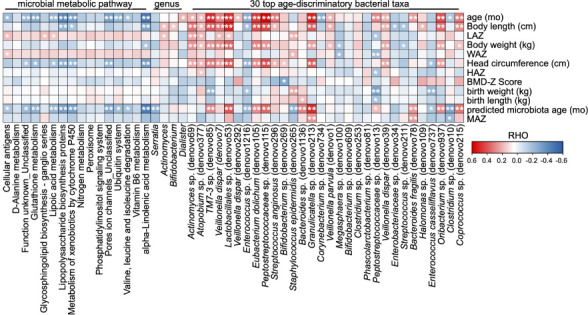
Correlations between the gut microbial taxa/functions and infant anthropometric features. The heatmap of the correlations between the significant altered gut microbial taxa/functions and the anthropometry features. Based on Spearman’s correlation coefficient, *p < 0.05, **p < 0.01.

We further analyzed the correlations between the above-mentioned significant changes in microbial taxa/functions and infant anthropometric parameters. The cellular antigens, glycosphingolipid biosynthesis-ganglio series, and lipoic acid metabolism were significantly positively related to LAZ, and glutathione metabolism, lipoic acid metabolism, lipopolysaccharide biosynthesis proteins, metabolism of xenobiotics by cytochrome P450, pore ion channels_unclassified, and alpha-linolenic acid metabolism exhibited significant negative correlations with infant head circumference (**Figure 5**). At the genus level, *Actinomyces* was significantly correlated with both body length and LAZ, while *Bifidobacterium* exhibited only a positive correlation with infant body length (**Figure 5**). At the OTU level, 14 of the 30 age-discriminatory OTUs were significantly positively related to body length, and four of them were also significantly correlated with LAZ (**Figure 5**). Twelve of the 30 age-discriminatory OTUs were significantly positively related to infant head circumference, and four of them were significantly related to body length and LAZ (**Figure 5**). *Enterococcus* sp. (denovo1216), *Enterobacteriaceae* sp. (denovo34), and *Streptococcus* sp. (denovo211) were negatively correlated with infant body length, while *Enterococcus* sp. (denovo1216) and *Enterococcus casseliflavus* (denovo737) were negatively correlated with infant head circumference (**Figure 5**). Notably, the body or head growth-negative-related OTUs were typically considered as potential pathogens clinically.

## Discussion

The acquisition and development of gut microbiota during the early human postnatal period plays a fundamental role in future somatic body (Subramanian et al., 2014; Raman et al., 2019) and nervous system (Seki et al., 2021) maturation. Serious failures in gut microbiota development or in other processes such as stunted gut microbiota typically cause or accompany infections (Shao et al., 2019), neurodevelopmental disorders (Seki et al., 2021; Wan et al., 2021; Lou et al., 2022), GI problems (Hayden et al., 2020), and malnutrition (Subramanian et al., 2014). During this period, individual gut microbiomes experience violent fluctuations (de Goffau et al., 2019; Kennedy et al., 2021) that can complicate pediatric clinical practice and, deconstruction and interpretation of gut microbiota is thus urgently needed. To our knowledge, this is the first study to focus on the gut microbiota profile in subjects with a clinical diagnosis of failure to thrive (FTT) and in preterm subjects with gestational age-corrected healthy growth (NFTT-pre). We observed that the gradually aggravated underdevelopment of gut microbiota from the NFTT-pre to FTT groups in both the profiles of colonization of normal bacteria and enrichment of microbial functions when compared to these characteristics in the NFTT group, and we determined that many of the significantly shifted microbial features (microbiota age, microbial taxa, and functions) were correlated with infant growth parameters (**Figure 1**).

Statistically, infant chronological age contributed to the most significant variance in gut microbiota, thus indicating the dynamics and complexity of human early life. In clinical applications, we must consider the understanding, application, and interpretation of the sequencing results of the gut microbiota corresponding to individual age. Additionally, feeding methods during the postnatal 12 months (particularly the feeding method during the 3-6 months) induced a second impact on gut microbiota that revealed the encouraging role of food selection in the future feeding and rebuilding of infant gut microbiota. Surprisingly, the head circumference of infants was also associated with considerable variance in gut microbiota (**Figure 2A**) and exhibited significant correlations with infant microbiota age and microbial taxa and functions (**Figure 1G**, **2A**, and **5A**), thus suggesting that the development of early gut microbiota is closely related to the development of the infant brain. Perinatal events such as assisted reproductive technology (Lu et al., 2020), mode of delivery (Nagpal et al., 2016; Shao et al., 2019), and premature delivery (Younge et al., 2018) have been recognized as interfering factors in the infant gut microbiota involved in subsequent growth (Rogers et al., 2020). We provided evidence that IUGR also significantly affects the infant gut microbiota.

Although after gestational age correction where the infant experienced a longer duration of exposure to the external environment and enteral nutrition, infants in the NFTT-pre group still exhibited disorganized development of gut microbiota that was more serious in the FTT group (**Figures 2B-D**). Premature birth caused abnormal gut microbiota development in the NFTT-pre group but did not aggravate the disruption of gut microbiota development in children with FTT (**Figure S2**), thus indicating that intrinsic host microenvironmental factors may dominantly drive (affect) infant microbiota development. More significant differences were observed in regard to the patterns of gut microbiota disorders in the NFTT-pre and FTT groups. At the ecosystem level, an increase in alpha diversity is an important component of infant gut microbiota growth (Stewart et al., 2018; Raman et al., 2019). Growth of the alpha diversity indices, the observed OTU, and the Shannon index were inadequate in the FTT group. From a microbial taxonomic perspective (**Figure 3**), unlike the *Firmicutes* and *Bacteroidetes* that predominated healthy adult gut, the phylum *Actinobacteria* was the predominant in infant gut in regard to its members *Bifidobacterium* and *Actinobacteria*. Trends of proportional change in *Actinobacteria* were inversed between the NFTT and FTT groups across age, and the relative abundance of *Bifidobacterium* was significantly decreased in subjects with FTT (**Figure 3C**). The loss of early intestinal-dominant bacteria and disorganized growth of age-discriminatory OTUs in FTT provided opportunities for the colonization of unexpected microorganisms. Therefore, we detected more untimely colonization and enrichment of the potential pathogens *Streptococcus* and *Enterococcus* in FTT (**Figures 3B, E**).

Our results regarding microbial functions further suggest potential interactions between the gut microbiota and host developmental homeostasis. When comparing each group, most of the FTT-perturbated microbial functions decreased and primarily belonged to group 1, the functional cluster within which the microbial functions were enriched from to 4-9 months of age (**Figures 4A-D**). Metabolites of these functions were considered to be involved in the process of cell growth, lipid metabolism, and synthesis of small molecular acids and exhibited correlations with microbial taxa and infant anthropometric features. For example, we observed that the bacterial pathway for lipoic acid metabolism was significantly increased in the NFTT group and was positively correlated with the LAZ of infants. Increased microbial lipoic acid metabolism in the gut can induce more lipoic acid to be absorbed by the infant through the intestine, and this may help the body to resist oxidative damage and produce more coenzymes to thereby benefit infant physical growth (positively related to both LAZ and MAZ).

In conclusion, we observed underdevelopment of the gut microbiota in infants with FTT in regard to both the profiles of microbial taxa colonization and microbial function enrichment. Future studies are required to test if restoration of altered microbial composition and functions such as increasing the relative abundance of *Bifidobacterium* or treating infant growth to defeat negatively related microbial metabolites with benefit infant growth and constitute a microecological perspective for both disease tracing and treatment.

## Data availability statement

Data are available upon reasonable request. All data relevant to the study are included in the article or are uploaded as supplementary information. All 16S rRNA raw data were submitted to the China National GeneBank Database, CNGBdb (accession number: CNP0002361).

## Ethics statement

The study was approved by Research Ethics Committee of Changsha Hospital for Maternal and Child Health Care (NO: 2020007). Written informed consent to participate in this study was provided by the participants’ legal guardian/next of kin.

## Author contributions

XK, JH, MZ, and TW conceived and devised the study; XK, TC, WZ, FZ, and SY collected the fecal samples and clinical data; XK, MZ, DM, and QM performed data analysis and drew figures. MZ and XK drafted the manuscript. XK and JH supervised the study and revised the manuscript accordingly. All authors critically reviewed the manuscript and approved its final version for submission.

## Supplementary material

Supplementary Figure 1 Predicted microbiota age of infants among groups. A. Mean values of the predicted microbiota age among each group and subgroup analysis according to infant chronological age. Mean values ± SEM are plotted. B. Microbiota for Age Z score (MAZ) in the FTT and NFTT-pre groups predicted using the Random-forest (RF) model. Mean values ± SEM are plotted. One-way ANOVA, *p < 0.05.

Supplementary Figure 2 No statistical difference in gut microbiota between FTT infants with or without preterm birth. Three-dimensional diagram of principal component analysis (PCoA) based on OTU-level Bray–Curtis distance. Plots of each sample were dyed according to group.
