# Supplementary figures and images for: Underdevelopment of gut microbiota in failure to thrive infants of up to 12 months of age
Source: Front Cell Infect Microbiol. 2022 Dec 12;12:1049201. doi: 10.3389/fcimb.2022.1049201 (PMC9791048; doi:10.3389/fcimb.2022.1049201)

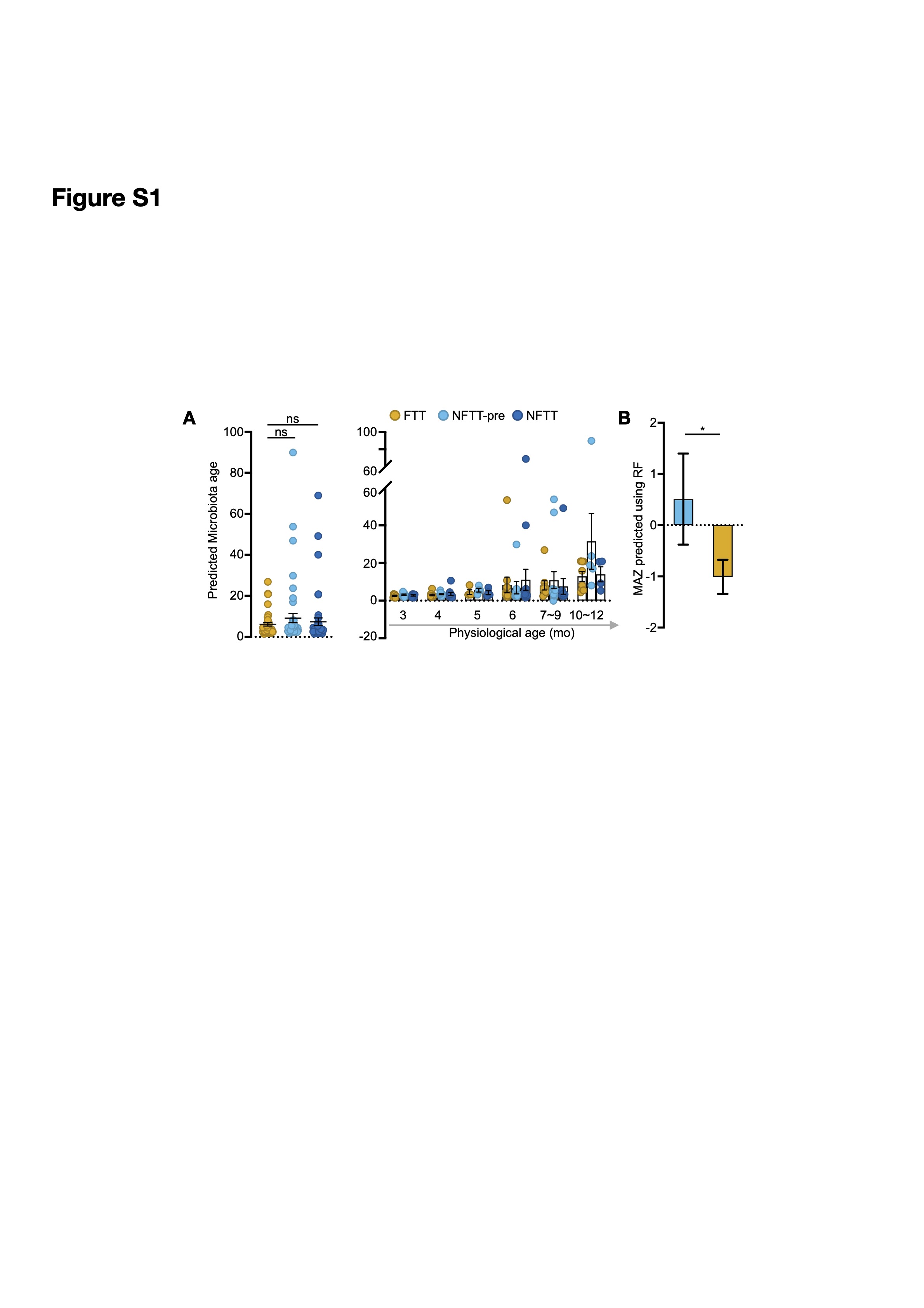

Supplement: Supplementary Figure 1 — Predicted microbiota age of infants among groups. A. Mean values of the predicted microbiota age among each group and subgroup analysis according to infant chronological age. Mean values ± SEM are plotted. B. Microbiota for Age Z score (MAZ) in the FTT and NFTT-pre groups predicted using the Random-forest (RF) model. Mean values ± SEM are plotted. One-way ANOVA, *p < 0.05. [file Image_1.jpeg]

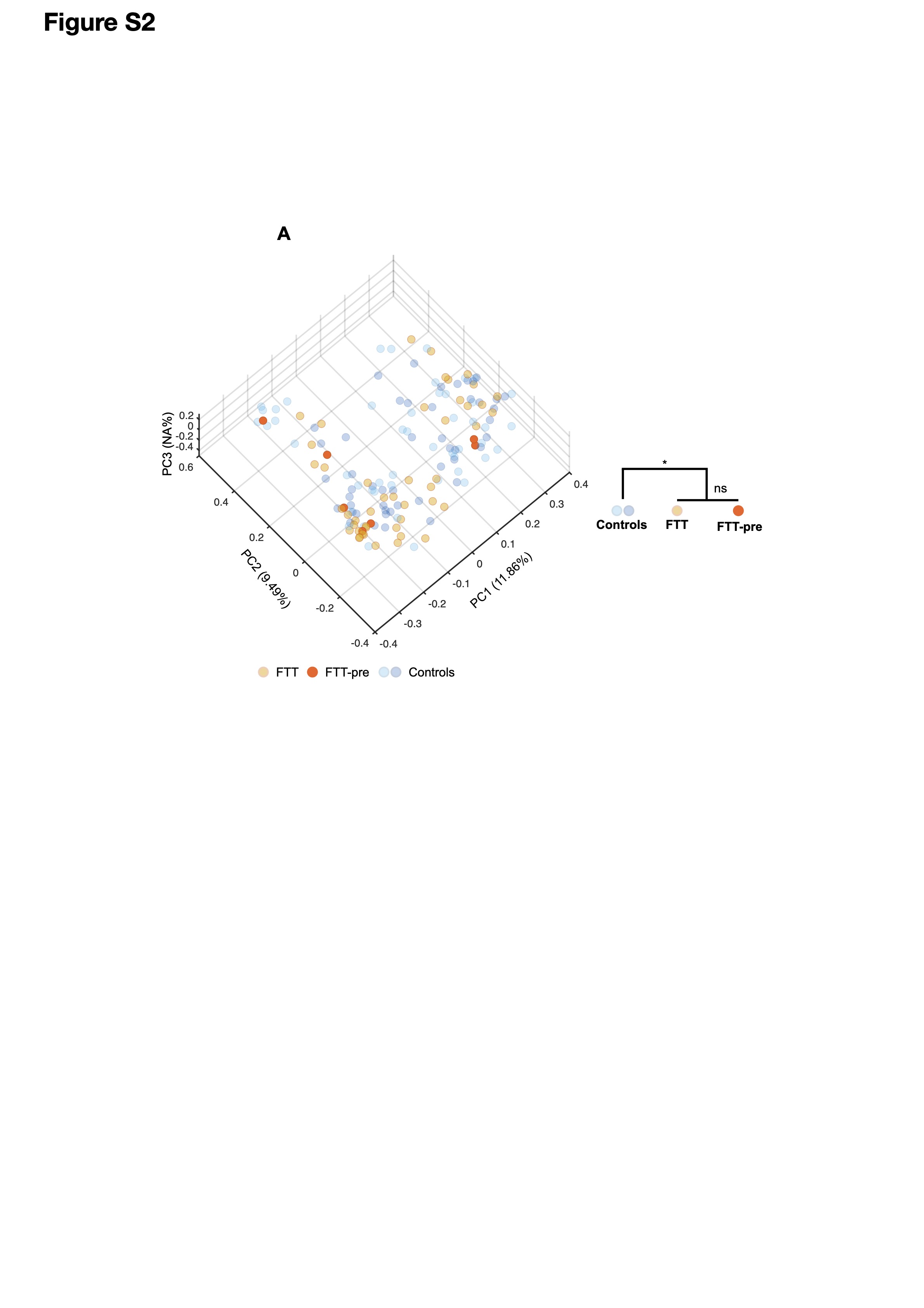

Supplement: Supplementary Figure 2 — No statistical difference in gut microbiota between FTT infants with or without preterm birth. Three-dimensional diagram of principal component analysis (PCoA) based on OTU-level Bray–Curtis distance. Plots of each sample were dyed according to group. [file Image_2.jpeg]
